# The Global Epidemiology of Impetigo: A Systematic Review of the Population Prevalence of Impetigo and Pyoderma

**DOI:** 10.1371/journal.pone.0136789

**Published:** 2015-08-28

**Authors:** Asha C. Bowen, Antoine Mahé, Roderick J. Hay, Ross M. Andrews, Andrew C. Steer, Steven Y. C. Tong, Jonathan R. Carapetis

**Affiliations:** 1 Menzies School of Health Research, Charles Darwin University, Darwin, Northern Territory, Australia; 2 Department of Infectious Diseases, Princess Margaret Hospital for Children, Perth, Western Australia, Australia; 3 Telethon Kids Institute, University of Western Australia, Perth, Western Australia, Australia; 4 Department of Dermatology, Hôpital Pasteur, Colmar, France; 5 International Foundation for Dermatology, London, United Kingdom; 6 Skin Infection Clinic, Kings College Hospital NHS Trust, Denmark Hill, United Kingdom; 7 Royal Children’s Hospital, Melbourne, Victoria, Australia; 8 Institute for Child Health Research, University of Melbourne, Victoria, Australia; 9 Department of Infectious Diseases, Royal Darwin Hospital, Darwin, Northern Territory, Australia; Wake Forest University School of Medicine, UNITED STATES

## Abstract

**Objective:**

We conducted a comprehensive, systematic review of the global childhood population prevalence of impetigo and the broader condition pyoderma.

**Methods:**

PubMed was systematically searched for impetigo or pyoderma studies published between January 1 1970 and September 30 2014. Two independent reviewers extracted data from each relevant article on the prevalence of impetigo.

**Findings:**

Sixty-six articles relating to 89 studies met our inclusion criteria. Based on population surveillance, 82 studies included data on 145,028 children assessed for pyoderma or impetigo. Median childhood prevalence was 12·3% (IQR 4·2–19·4%). Fifty-eight (65%) studies were from low or low-middle income countries, where median childhood prevalences were 8·4% (IQR 4·2–16·1%) and 14·5% (IQR 8·3–20·9%), respectively. However, the highest burden was seen in underprivileged children from marginalised communities of high-income countries; median prevalence 19·4%, (IQR 3·9–43·3%).

**Conclusion:**

Based on data from studies published since 2000 from low and low-middle income countries, we estimate the global population of children suffering from impetigo at any one time to be in excess of 162 million, predominantly in tropical, resource-poor contexts. Impetigo is an under-recognised disease and in conjunction with scabies, comprises a major childhood dermatological condition with potential lifelong consequences if untreated.

## Introduction

Impetigo is a common dermatosis of childhood. Recent estimates of the global burden of impetigo are 111 million children from developing countries [1] to 140 million [2, 3] people affected at any one time. However, these estimates were based on a limited literature review of impetigo in the context of larger studies, have not been recently updated and acknowledge that impetigo estimates are imprecise due to the paucity of published literature from the highest prevalence contexts.[1–3] Additionally, most available data arise from hospital records, which may under-represent the true population prevalence of skin disease due to selection bias [4–9] and minimal health-care seeking for skin diseases in resource-poor contexts. [10–12] In this new study we have systematically collated data and updated estimates of the global population prevalence of childhood impetigo.

*Staphylococcus aureus* and *Streptococcus pyogenes* cause superficial skin infections. [13] Pyoderma is used to describe all superficial bacterial skin infections associated with the production of pus and is inclusive of impetigo (also known as skin sores or school sores), ecthyma and furunculosis. [14] Here we include reports on all pyodermas, but with a particular emphasis on impetigo. In resource-poor communities impetigo maintains a high burden of disease and affects well-being.[15] In addition, the infectious (staphylococcal and streptococcal cellulitis, bacteraemia, and deep tissue infections) and post-infectious (glomerulonephritis, rheumatic fever) sequelae cause high and persistent morbidity [1, 16–18] and variable mortality. [19, 20] The relative cost to families of missed school days and procuring treatments that may or may not work is also high. [10, 21]

The aim of this systematic review is to evaluate the prevalence of impetigo from studies in the general population (community or school surveys) and to explore variations in the epidemiology.

## Methods

### Search Strategy

This systematic review is reported according to PRISMA guidelines. [22] References were identified through PubMed for papers published in English between January 1970 and September 2014, which reported population based studies of skin disorders, with specific reference to impetigo or pyoderma prevalence. The search terms used were ["impetigo" OR "pyoderma"] AND ["Africa" OR "Asia" OR “Latin America” OR “Pacific” OR “Oceania” OR “North America” OR “Europe” OR “Russia” OR “China” OR “India” OR “Developing Country” OR “tropical” OR “Indigenous”]. Duplicates were removed before titles were reviewed for relevance (epidemiology, prevalence, impetigo, and pyoderma). If the title contained insufficient detail, abstracts or entire articles were reviewed for pre-determined inclusion criteria. The bibliographies of retrieved papers were hand-searched for additional references. An extensive search of the grey literature did not add any additional relevant studies after the abstracts or entire article was reviewed.

### Selection Criteria

Population-based, prevalence studies, with extractable data on children with pyoderma or impetigo were included if a physical examination by a clinician was performed. Wherever a term was used that inferred a bacterial skin infection (pyoderma, impetigo or sores) and numerator and denominator or proportion-affected data were available, these have been reported. Outpatient dermatology clinic and hospital-based studies from developing countries were excluded. Due to the overlap between impetigo and scabies, data on scabies was also extracted where available.

### Reviewer Assessment

Papers meeting the inclusion criteria were sourced in full-text and data extracted by two reviewers independently. All papers were assessed by AB and a subset by each of the co-authors, after determining that they were not an author or involved in the primary data collection of any of the studies. Extracted data included date, country, climate, rural/urban environment, study site (e.g. school, household), study design, sampling method, population, age range, gender, qualifications of person conducting the screening, case definition, definition of bacterial skin infection, number of participants, number with impetigo, childhood and adult prevalence, location of lesions, microbiology and presence of scabies.

### Definitions

Where the study date was not reported, the year of publication was used. The sampling method was defined as exhaustive if ≥85% of available population were surveyed and non-exhaustive if it was below 85%. Other options for sampling method included convenience (non-random selection of the available population), targeted (orphanages or institutions) and random selection of participants. The definition for a bacterial skin infection was recorded and later categorised as pyoderma (if the text used this term or indicated impetigo, folliculitis, ecthyma, furunculosis, and cellulitis), impetigo (only impetigo or skin sores were studied) and secondarily infected scabies (the primary focus of the study was scabies with a secondary focus on bacterial skin infection). We have concentrated on reporting impetigo prevalence in children. Where available, adult data were incorporated. If children and adults were included in the study, but separate rates were not provided, then the community wide prevalence of impetigo was reported. It was not possible to ascertain from the studies how representative the study sample was of the broader population.

Countries were categorised into regions according to the United Nations (UN) Population Division (www.esa.un.org/wpp/excel-Data/country-Classification.pdf, last accessed 10 November 2014). Childhood was defined as ages 0 to 15 years. To assess the total population at risk of impetigo based on the median prevalence estimate, population data from the UN Department of Economic and Social Affairs Population Division for 2012 were used, (www.esa.un.org/unpd/wpp/unpp/panel_indicators.htm, last accessed 7 December 2014). Each country was also categorised according to the World Development Index as at July 2005 (www.data.worldbank.org) as high (>$US 10,725 gross national income (GNI) per capita), upper middle ($US 3,466–$10,725 GNI per capita), lower middle ($US 876–$3,465 GNI per capita) or low (≤$US 875 GNI per capita) income. The Koppen Climate Classification System is the most widely used classification of climates and recognizes five major climate systems, tropical, arid, temperate, cold and polar. [23] Where climate data was not reported, the Koppen classification was used to code the climate by country and region.[23]

### Statistical Analysis

The data are synthesized into a narrative summary. Statistical analysis was performed using Stata13 (Statacorp, Texas, USA). Where prevalence estimates have been combined to understand regional and global burden of impetigo, the median prevalence has been used. In order to estimate regional and global burden of impetigo, the median prevalence has been applied to the 2011 Australian census and 2012 United Nations population estimate for less developed countries. The metaregression command in Stata was used to calculate the pooled prevalence (random-effects model), using the inverse of the sample size to account for variability in study size. To assess for any variation in reporting of prevalence based on the use of either pyoderma or impetigo, we assessed the use of each term, and calculated a statistical difference between the median prevalence using a chi squared statistic.

## Results

**Fig 1:**
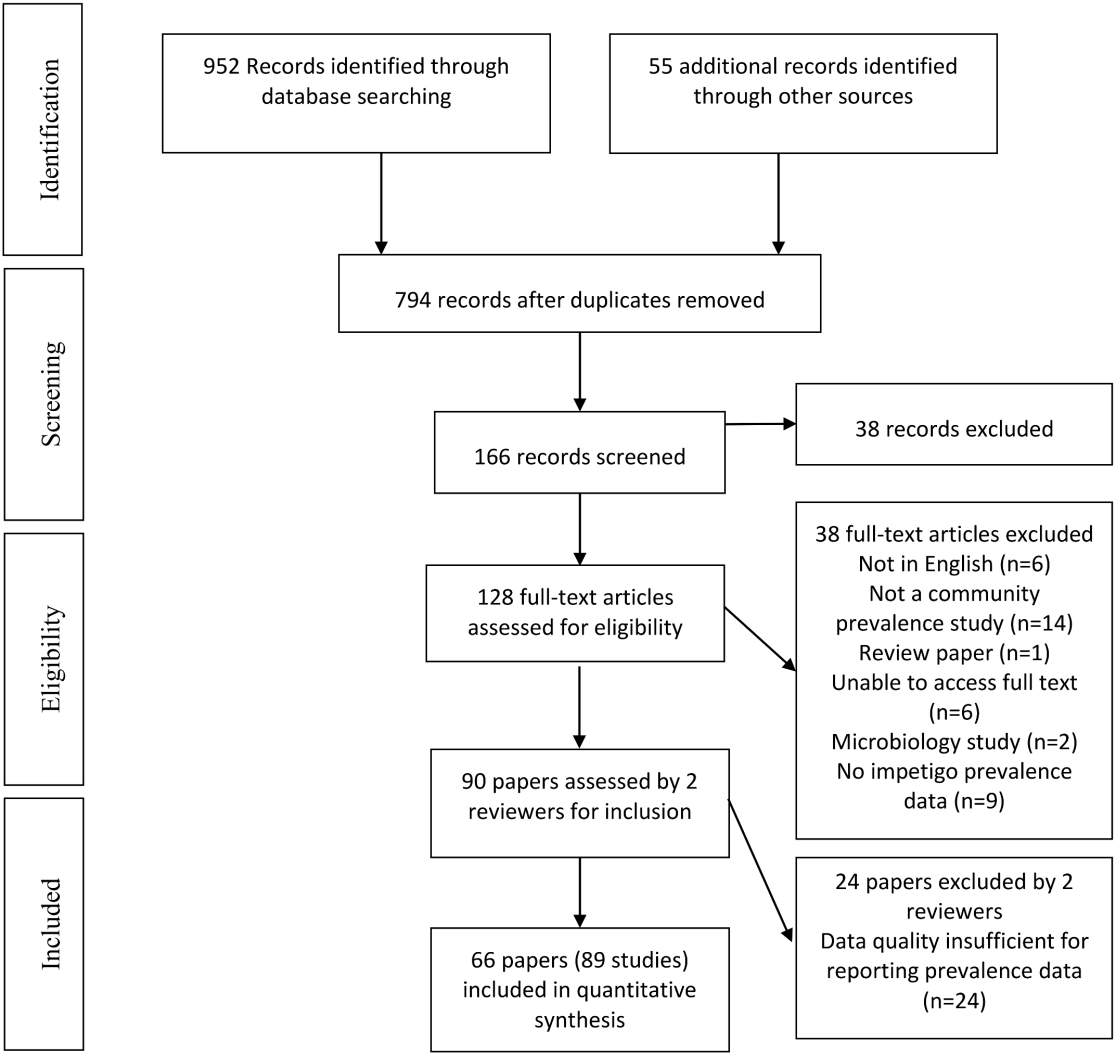
Flowchart of systematic review according to the PRISMA statement.

Of the 1007 titles identified, 952 were from database searching and 55 from additional sources (Fig 1). Two hundred and thirteen duplicate records were removed and 628 papers excluded, due to insufficient data on impetigo or because the studies were conducted in dermatology clinics or hospitals. Full text review of the 128 remaining records resulted in 38 further exclusions, leaving 90 papers. A further 24 were excluded due to insufficient data reported on impetigo prevalence. The final dataset includes 66 papers [4, 6–9, 15, 24–82] reporting on 89 studies (Fig 1, Table 1, S1 Table) conducted over a 45-year period.

**Table 1:** Number of studies of impetigo prevalence by decade, country and region.

Decade	Number of studies	Countries	Regions
1970–1979	28	Colombia, Ghana, Tanzania, New Zealand, Brazil, India, USA, Gambia, Panama	Latin America & Caribbean, Africa, Asia, Oceania, North America
1980–1989	15	Pakistan, Solomon Islands, Nigeria, Ethiopia, Vanuatu, India, Fiji, Canada	Asia, Oceania, Africa, North America
1990–1999	20	Australia, Honduras, Mali, Malaysia, Ethiopia, Tanzania, Ecuador, Samoa, Taiwan, Kenya, Solomon Islands	Oceania, Latin America & Caribbean, Africa, Asia
2000–2009	23	Nepal, Australia, India, Fiji, Tanzania, Nigeria, Timor Leste, Turkey, Mali, Ghana, Gabon, Rwanda, Egypt	Asia, Oceania, Africa
2010–2014	3*	Ethiopia, Cameroon, Tanzania	Africa

* Two studies were published in 2010 and did not provide a year of data collection in the manuscript.

The studies were predominantly from Africa (30/89, 34%), Asia (20/89, 22%) and Oceania (19/89, 21%) and represented populations from 31 countries (Table 1). Studies with data available by decade were: 1970s (28, 32%), 1980s (15, 17%), 1990s (20, 23%), 2000s (23, 26%) and since 2010 (3, 3%).

Data on impetigo prevalence were available for 174,508 individuals of whom 145,028 were children. The study size varied, ranging from 31 to 19,775 participants per study. The median study size was 636 participants (inter-quartile range [IQR] 305–1817), median prevalence 11·2% (IQR 4·2–19·4%) and pooled prevalence 15·5% (95% CI 12·1–19·0%) (Table 2). Extractable prevalence data were available on children in 82 (92%) of the studies. The median impetigo prevalence in children was 12·3%, (IQR 4·2–19·3%) and pooled prevalence 16·6% (95% CI 12·7–20·5%). The median number of children in each study was 534 (IQR 258–1,729).

**Table 2:** Summary statistics of available studies by age grouping.

	Total available population (N = 89 studies)	Childhood population (N = 82 studies)	Adult population (N = 11 studies)
**Median (IQR) prevalence of impetigo**	11·2% (4·2–19·4%)	12·3% (4·2–19·4%)	4·9% (3·1–9·6%)
**Pooled prevalence (95% CI)**	15·5% (12·1–19·0%)	16·6% (12·7–20·5%)	9·7% (2·2–7·2%)
**Median (IQR) number of participants per study**	636 (305–1,817)	534 (258–1,729)	638 (264–1,645)
**Population with impetigo**	23,759	19,811	2,427
**Total population studied**	174,508	145,028	18,246

Reported impetigo prevalence ranged from 0·2% to 90%. The highest median prevalence of childhood impetigo was reported from Oceania, where from 19 studies, the median prevalence was 40·2% (IQR 17·2–48·1%). Excluding studies from Australia and New Zealand, of the eight remaining studies from Oceania, the median prevalence remained high at 29·7% (IQR 14·7–42·0%). The median impetigo prevalence in Africa was 7% (IQR 4·1–12·3%), Asia 7·3% (IQR 3·0–16·1%), resource poor populations in North America 13·3% (IQR 2·1–19·4%) and Latin America and the Caribbean 15·5% (IQR 12·2–20·8%). There was no data available for Europe or China.

We estimated the burden of impetigo in low and low-middle income countries using estimates for the global population below 15 years of age resident in less developed countries in the years 2000–2009 to be at least 1.6 billion children. Utilising the median impetigo population prevalence of 9·9%, (IQR 4·1–16·6%) from the studies conducted in developing economies (WDI2005 index of lower-middle or low) since 2000, the estimated population with impetigo at any one time is more than 162 million children. Excluding China where no studies were available, reduces the estimate to 137 million children with impetigo, in low and low-middle income countries. Table 3 outlines the regional estimates of children with impetigo at any one time using the available data.

**Table 3:** Estimates of children with impetigo by regions of the world with available data*.

Region	Population in 2012 under 15 years	Median impetigo prevalence in children	Estimated number of children with impetigo
Africa	424,072,000	7% (IQR 4·1–12·3%)	29,685,040
Asia	1,060,076,000	7·3% (IQR 3·0–16·1%)	77,385,548
Oceania*	3,653,000	29·7% (IQR 14.7–42·0%)	1,084,941
Latin American & Caribbean	167,654,000	15·5% (IQR 12·2–20·8%)	25,986,370
**TOTAL**	**1,655,455,000**	**N/A**	**134,141,899**

*Studies from Australia, New Zealand and North America excluded as all these studies were conducted in small, impoverished populations within these countries that may not reflect the overall burden of impetigo for the childhood population.

There were 10 population prevalence studies available for Australia. All reported data from children living in remote Indigenous communities of northern Australia, with no studies available for non-Indigenous children. The median prevalence reported from these studies was 44·5% (IQR 34·0–49·2%). Four studies were conducted since 2000, with a median prevalence of 43·0% (IQR 40·2–45·7%). We estimated the total number of remote Indigenous children with impetigo at any one time by applying the median prevalence of 44·5% to the remote living Indigenous population from the states of Western Australia, Queensland and Northern Territory aged less than 15 years in the 2011 Australian census (35,272). We estimate 15,696 Indigenous children are suffering from impetigo at any one time. This is the first time that prevalence estimates have been used to generate a total number at risk amongst Australian Indigenous children. This will be important for local health care planning.

Only 13 (15%) studies reported prevalence of impetigo according to age group. Of these, the median prevalence in 0–4 year olds was 19% (IQR 15–31%), 5–9 year olds 19% (IQR 12–43%) and 10–14 year olds 10% (IQR 7–28%). Most studies, 58/89 (65%) (Table 4), were from low or low-middle income countries. The remainder were from middle-income or resource-poor populations within high-income countries. Table 5 summarises the median prevalence estimates according to income level of the country, with the highest estimates coming from underprivileged populations within high-income countries.

**Table 4:** Classification of studies by region and World Bank Development Indicator in 2005.

Region	High income	Upper Middle income	Low Middle Income	Low Income
Oceania	Australia (10) New Zealand (1)		Fiji* (4) Vanuatu (1) Samoa (1)	Solomon Islands* (2)
Africa		Gabon (1)	Egypt* (2)	Ghana* (3) Rwanda (1) Cameroon* (1) Ethiopia (3) Nigeria* (2) Tanzania (9) Kenya (3) Mali (3) The Gambia (2)
Asia	Taiwan (1)	Malaysia* (2) Turkey* (1)		India* (12) Nepal (2) Pakistan* (1) Timor-Leste* (1)
Caribbean & Latin America		Panama* (2)	Honduras* (1) Brazil (2)* Colombia* (1) Ecuador*(1)	
North America	Canada (6) USA (7)			
**TOTAL**	**25**	**6**	**13**	**45**

*Category has shifted rather than remaining stable within period from 1987–2013.

**Source:** data.worldbank.org/data-catalog/world-development-indicators, accessed 12.11.2014.

**Table 5:** Median prevalence of impetigo in childhood and overall, categorised by the World Development Index.

WDI and number of studies	Median childhood prevalence (IQR) N = 82	Median overall prevalence (IQR) N = 89
**High income (N = 25)**	19·4% (IQR 3·9–43·3%)	19·4% (IQR 3·9–43·3%)
**Middle income (N = 4 [childhood] OR N = 6 [overall])**	9·9% (1·8–18·6%)	9·9% (IQR 2·0–15·1%)
**Low-Middle (N = 12)**	14·5% (IQR 8·3–20·9%)	12·8% (7·8–18·3%)
**Low (N = 41 [childhood] OR N = 46 [overall])**	8·4% (IQR 4·2–16·1%)	7·9% (4·3–16·1%)

Fifty-seven (64%) studies also reported data on scabies prevalence. The median prevalence of scabies was 3·3% (IQR 0·7–12·9%). Twenty-seven (87%) countries had available data on scabies prevalence. Scabies prevalence varied by region with the highest median prevalence found in Oceania (n = 14) at 16% (IQR 4·9–25%). The median scabies prevalence in Africa (n = 28) was 2% (IQR 0·7–7%) and in Asia (n = 11) was 3·4% (IQR 0·9–11·9%). The median prevalence of scabies in studies from Latin America and the Caribbean (n = 3) was 3% (IQR 0.6–10%). Scabies prevalence also varied by decade. In the 1970s (n = 12), the median was 3·2% (IQR 1·4–13%), 1980s (n = 8) 1·1% (IQR 0·4–1·8%), 1990s (n = 14) 9·2% (IQR 4·9–17%), 2000s (n = 20) 1·9% (IQR 0·6–15·1%) and since 2010 (n = 3) 1·4% (IQR 0·3–1·8%). The prevalence of pyoderma and scabies were closely correlated (p = 0·01).

Thirty-four (38%) studies reported culture based microbiology results, predominantly on streptococcal infection (n = 31). Only 11/89 (12%) studies reported on the relative contributions of *S*. *pyogenes* and *S*. *aureus* from microbiological culture of skin lesions. *S*. *pyogenes* was identified in a median of 74% (IQR 57–95%) of cultures and *S*. *aureus* in a median of 64% (IQR 53–80%) of cultures. These culture results should be treated with caution as the microbiology methods were heterogenous or not reported.

Data were reported on body distribution of impetigo in 23 studies, with the lower limbs being the most common site in 21 studies (91%). In 11 studies, the proportion of body regions affected was given. After re-classification as lower limbs, upper limbs and other [scalp, face, neck, torso], the medians for body region distributions (these were not mutually exclusive) were 58% (IQR 44–86%), 18% (IQR 14–54%) and 38% (IQR 5–43%) respectively.

Studies were classified broadly as rural (n = 61), urban (n = 15) or both (n = 13). A higher prevalence of impetigo was reported from rural locations compared to urban settings (Table 6). Most studies were from tropical environments, 67/89 (75%) with the remainder from cold 9/89 (10%), temperate 9/89 (10%) and arid 4/89 (5%) climates. Median impetigo prevalence in childhood was 12·2% (IQR 4·8–20·8%), 17·5% (IQR 8·2–42·8%), 15·8% (IQR 2·2–30·2%) and 3·9% (IQR 1·0–13·3%) for tropical, arid, temperate and cold climates respectively.

**Table 6:** Variability in median impetigo prevalence by urban and rural study locations.

	Median impetigo prevalence overall	Median impetigo prevalence in children
Rural	N = 61 studies	N = 55 studies
	13·3% (6·7–20·9%)	16·1% (5·9–22·6%)
Urban	N = 15 studies	N = 14 studies
	4·8% (2·0–10·0%)	4·5% (2·0–7·3%)
Both	N = 13 studies	N = 13 studies
	5·8% (2·4–13·3%)	5·8% (2·4–13·3%)

There was variability in the population sampling techniques employed. The most common technique described was exhaustive sampling. Of the exhaustive population surveillance studies (n = 57/89, 64%), the median number of participants was 636 (IQR 305–2528) and median prevalence 10·8% (IQR 4·3–19·4%). The median prevalence was 4% (IQR 2·0–16·6%) in the seven studies where random sampling was employed.

Twenty-seven (30%) studies reported on impetigo as the primary definition under investigation. Of these, 25 have data on the childhood prevalence of impetigo, median 13·3% (IQR 2·3–22·6%). Pyoderma was reported in 62 (70%) studies with a median prevalence of 11·8% (IQR 5·1–19·0%). There was no statistical difference in the median prevalence reported based on these definitions, p = 0·85. Both definitions were used throughout all decades of the study. There was some variability in the use of definition by region: studies from Africa, Asia and Latin America predominantly reported on pyoderma, whereas studies from Oceania used either definition and North American studies were more likely to report on impetigo (Table 7).

**Table 7:** Regional variation in the use of pyoderma or impetigo to describe bacterial skin infections.

Region	Pyoderma reported (%)	Impetigo reported (%)
Africa (N = 30)	27 (90%)	3 (10%)
Asia (N = 18)	15 (79%)	4 (21%)
Oceania (N = 19)	10 (53%)	9 (47%)
North America (N = 13)	3/13 (23%)	10/13 (77%)
Latin America & Caribbean (N = 7)	7/7 (100%)	0

## Discussion

This systematic review provides comprehensive data and confirms an ongoing, high burden of impetigo in childhood, estimating more than 162 million children in low and low-middle income countries are affected at any one time. Our study revises upwards the previous point-prevalence estimate of 111 million children with impetigo.[1] The reported burden has remained high throughout the study period, with a median prevalence of 12·3% (IQR 4·2–19·3%). Our estimate derived from 89 studies over 45 years is higher than previously published estimates, which were between 5 and 10%. [12] Impetigo is more than a benign, nuisance condition and these numbers demonstrate the public health priority of impetigo. Each study is valuable in describing the impetigo burden for a local or regional population, but collectively they tell a far more compelling story of an under-appreciated disease.

The data cover large regions of the globe and include studies from countries that were not available in previous disease burden estimates. By highlighting the global burden, which has previously been estimated at >2% of the global population at any one time, [3] an agenda for screening, treatment and further work can crystallise. This will inform primary prevention of kidney and heart disease and gram-positive bacterial sepsis in resource poor contexts. Likewise, highlighting the population-based burden in resource-poor settings may prioritise the conduct of treatment studies in contexts with the highest burden of impetigo. The Cochrane review on the optimal treatment of impetigo[83] includes studies predominantly from high income countries and references only one study (out of 68) conducted in a similar setting to those reported in our systematic review.

Collectively, the data describe limited progress in impetigo control. Over a 45-year interval, the burden of impetigo has remained relatively unchanged. Adding bacterial skin infections to the list of neglected tropical diseases is one strategy that might accelerate progress. We also describe a high impetigo burden in impoverished populations within wealthy countries, in keeping with version 2.0 of the neglected tropical diseases agenda where ‘blue marble health’ identifies the contribution of poverty in all countries as a key underlying factor of neglected tropical diseases. [84]

Our study confirms that the greatest burden of impetigo is in children, with steady decreases in prevalence with increasing age. [12, 32] We also confirm childhood impetigo predominantly affects the lower limbs in these studies, and is caused by both *S*. *pyogenes* and *S*. *aureus*. Despite our study reflecting predominantly impoverished settings, increases in impetigo have also been reported in children in developed countries attending general practices for care [85] and it causes a large volume of health care consultations in all regions of the world. [86–88]

The definitions and clustering of conditions used was variable. Using the two dominant terms of impetigo and pyoderma, there was no statistical difference in the median prevalence, suggesting that our inclusion of both terms as a descriptor of bacterial skin infections in populations is robust. Other forms of pyoderma were much less common which may explain the similarities in population prevalence of impetigo and pyoderma.

We recommend that guidelines for skin surveillance be used for future studies. [89] Our study found the clinical skills of those performing cutaneous disease surveillance varied greatly, ranging from dermatologists to community health workers trained in identification of skin conditions. This limitation may have resulted in under-reporting of impetigo. In addition, many of the studies were focussed on a specific dermatosis, conducted in the context of nutrition or child health surveys or to demonstrate the substantial burden of skin disease and significant unmet need. [80]

It is possible that studies have been conducted in the regions of highest disease burden leading to an over-estimate of the global burden. [12] We think this is unlikely, given the size, consistency and duration of the estimated burden of impetigo that we have described. However, population-based prevalence studies from Europe, East and South-East Asia and more recent work from North America, are under-represented. The inclusion of geographic locations as search terms was used to explore possible publication bias. We were not able to identify additional studies using this approach. Despite these gaps, our findings are consistent with the 2010 global burden of disease estimates for impetigo. [2, 3] Our study may also be limited by the exclusion of unpublished data and by restricting the analysis to English language publications. The grey literature was searched without adding to the studies synthesised.

## Conclusions

Impetigo prevalence throughout was highest in Oceania, in both resource-poor countries and underprivileged populations within high-income countries. This synthesis of studies is important for regional and national targeting of healthy skin interventions, as the burden of disease is large. At a global level, this study revises our estimate upwards of the number of children affected with impetigo at any one time from 111 million [1] to 162 million. This finding alone should drive a comprehensive public health and research agenda for the detection, treatment and prevention of impetigo in resource-poor contexts, and ongoing evaluation of comprehensive programs to train primary care workers in the treatment of skin infections. As antibiotics are the backbone of current treatment for impetigo, this disease burden may contribute to burgeoning antibiotic resistance in the absence of evidence-based treatment algorithms. [90] This combination of high prevalence and moderate morbidity makes impetigo a high population health priority.

## Supporting Information

S1 Table Overall and childhood pyoderma and scabies prevalence from 89 studies. *Resource-poor populations within high-income OECD countries, ^reflects year of publication when year study was commenced is unknown.
(DOC)
