# Supplementary material for: The Global Epidemiology of Impetigo: A Systematic Review of the Population Prevalence of Impetigo and Pyoderma
Source: PLoS One. 2015 Aug 28;10(8):e0136789. doi: 10.1371/journal.pone.0136789 (PMC4552802; doi:10.1371/journal.pone.0136789)
Supplement: S1 Table — *Resource-poor populations within high-income OECD countries, ^reflects year of publication when year study was commenced is unknown. (DOC) [file pone.0136789.s001.doc]

Supplementary Table

Overall and childhood pyoderma and scabies prevalence from 89 studies.

|  | | | **Overall** | | | | **Children (0—15 years)** | | | |  |
| --- | --- | --- | --- | --- | --- | --- | --- | --- | --- | --- | --- |
| **Country** | **Year** | **Number studied** | | **Number with pyoderma** | **Pyoderma prevalence (%)** | **Number studied** | | **Number with pyoderma** | **Pyoderma prevalence** | **Scabies prevalence** | |
| India[1] | 1967 | 488 | | 49 | 10.0 | 488 | | 49 | 10.0 | NS | |
| USA*[2] | 1969 | 39 | | 16 | 41.0 | 39 | | 16 | 41.0 | NS | |
| USA*[3] | 1970 | 444 | | 235 | 52.9 | 444 | | 235 | 52.9 | NS | |
| USA*[2] | 1970 | 31 | | 6 | 19.4 | 31 | | 6 | 19.4 | NS | |
| Colombia[4] | 1971 | 1,269 | | 169 | 13.3 | 1,269 | | 169 | 13.3 | NS | |
| USA*[2] | 1971 | 80 | | 12 | 15.0 | 80 | | 12 | 15.0 | NS | |
| Panama[5] | 1972 | 892 | | 135 | 15.1 | 628 | | 111 | 17.7 | 0.60% | |
| Panama[5] | 1972 | 131 | | 17 | 13.0 | NS | | NS | NS | NS | |
| India[6] | 1973 | 6,641 | | 392 | 5.9 | 6,641 | | 392 | 5.9 | NS | |
| Tanzania[7] | 1973 | 532 | | 37 | 7.0 | 532 | | 37 | 7.0 | 31% | |
| New Zealand*[8] | 1974 | 453 | | 6 | 1.3 | 453 | | 6 | 1.3 | 0 | |
| India[6] | 1974 | 1,038 | | 176 | 17.0 | 1,038 | | 176 | 17.0 | Common | |
| Brazil[9] | 1974 | 9,955 | | 1211 | 12.2 | 9,955 | | 1,211 | 12.2 | 3% | |
| Ghana[10] | 1975 | 3,770 | | 731 | 19.4 | NS | | NS | NS | NS | |
| Tanzania[11] | 1975^ | 794 | | 38 | 4.8 | 794 | | 38 | 4.8 | 12.90% | |
| Tanzania[11] | 1975^ | 305 | | 22 | 7.2 | 305 | | 22 | 7.2 | 0.70% | |
| Tanzania[11] | 1975^ | 120 | | 8 | 6.7 | 120 | | 8 | 6.7 | 3.30% | |
| Tanzania[11] | 1975^ | 636 | | 60 | 9.4 | 636 | | 60 | 9.4 | 31.60% | |
| Brazil[12] | 1976 | 775 | | 87 | 11.2 | 307 | | 64 | 20.8 | NS | |
| India[13] | 1976 | 89 | | 11 | 12.4 | 89 | | 11 | 12.4 | NS | |
| India[14] | 1976 | 19,775 | | 586 | 3.0 | 19,775 | | 586 | 3.0 | NS | |
| Gambia[15] | 1976 | 974 | | 87 | 8.9 | 332 | | 58 | 17.5 | 2.50% | |
| USA*[16] | 1976 | 593 | | 79 | 13.3 | 593 | | 79 | 13.3 | NS | |
| USA*[16] | 1976 | 434 | | 63 | 14.5 | 434 | | 63 | 14.5 | NS | |
| India[17] | 1977 | 1,890 | | 79 | 4.2 | 1,890 | | 79 | 4.2 | 8.20% | |
| Gambia[15] | 1977 | 945 | | 68 | 7.2 | 387 | | 39 | 10.1 | 2.00% | |
| USA*[16] | 1977 | 535 | | 104 | 19.4 | 535 | | 104 | 19.4 | NS | |
| India[18] | 1978 | 2,771 | | 59 | 2.1 | 1,509 | | 48 | 3.2 | 13% | |
| Pakistan[19] | 1980 | 444 | | 86 | 19.4 | 444 | | 86 | 19.4 | 1.90% | |
| India[20] | 1982 | 4,133 | | 431 | 10.4 | NS | | NS | NS | 0.12% | |
| Nigeria[21] | 1983 | 2,407 | | 87 | 3.6 | 2,407 | | 87 | 3.6 | NS | |
| Fiji[22] | 1983^ | 61 | | 14 | 23.0 | 61 | | 14 | 23.0 | 33% | |
| Canada*[23] | 1984 | 258 | | 2 | 0.8 | 258 | | 2 | 0.8 | NS | |
| Canada*[23] | 1984 | 378 | | 8 | 2.1 | 378 | | 8 | 2.1 | NS | |
| Solomon Islands[24] | 1984 | 10,224 | | 4,370 | 42.7 | 5,160 | | 2678 | 51.9 | 1.30% | |
| Canada*[23] | 1985 | 239 | | 10 | 4.2 | 239 | | 10 | 4.2 | NS | |
| Canada*[23] | 1985 | 202 | | 2 | 1.0 | 202 | | 2 | 1.0 | NS | |
| Canada*[23] | 1985 | 336 | | 13 | 3.9 | 336 | | 13 | 3.9 | NS | |
| Canada*[23] | 1985 | 334 | | 2 | 0.6 | 334 | | 2 | 0.6 | NS | |
| India[25] | 1986^ | 3,697 | | 39 | 1.1 | 3,697 | | 39 | 1.1 | 0.50% | |
| India[26] | 1988 | 666 | | 107 | 16.1 | 666 | | 107 | 16.1 | 0.90% | |
| Vanuatu[27] | 1989 | 18,223 | | 2133 | 11.7 | 9,569 | | 1537 | 16.1 | 16% | |
| Ethiopia[28] | 1989 | 1,842 | | 417 | 22.6 | 1,842 | | 417 | 22.6 | 1.70% | |
| Australia*[29] | 1990 | 120 | | 52 | 43.3 | 120 | | 52 | 43.3 | NS | |
| Tanzania[30] | 1991 | 936 | | 178 | 19.0 | NS | | NS | NS | 5.98% | |
| Honduras[31] | 1992 | 206 | | 43 | 20.9 | 206 | | 43 | 20.9 | 10% | |
| Ethiopia[32] | 1992 | 112 | | 6 | 5.4 | 112 | | 6 | 5.4 | 17% | |
| Australia*[33] | 1992^ | 180 | | 31 | 17.2 | 180 | | 31 | 17.2 | NS | |
| Australia*[34] | 1993 | 583 | | 198 | 34.0 | 583 | | 198 | 34.0 | NS | |
| Mali[35] | 1993 | 1,817 | | 224 | 12.3 | 1,817 | | 224 | 12.3 | 4.30% | |
| Kenya[36] | 1993 | 5,780 | | 735 | 12.7 | 5,780 | | 735 | 12.7 | 8.30% | |
| Ecuador[37] | 1993 | 495 | | 22 | 4.4 | 495 | | 22 | 4.4 | NS | |
| Tanzania[38] | 1994 | 1,114 | | 18 | 1.6 | NS | | NS | NS | 4.50% | |
| Australia*[39] | 1994 | 81 | | 39 | 48.1 | 81 | | 39 | 48.1 | NS | |
| Australia*[40] | 1994 | 126 | | 62 | 49.2 | 62 | | 43 | 69.4 | 28.80% | |
| Kenya[36] | 1995 | 4,358 | | 470 | 10.8 | 4,358 | | 470 | 10.8 | 10.50% | |
| Nepal[41] | 1995 | 458 | | 76 | 16.6 | 458 | | 76 | 16.6 | NS | |
| Australia*[42] | 1995 | 79 | | 71 | 89.9 | 79 | | 71 | 89.9 | 23% | |
| Malaysia[43] | 1995^ | 41 | | 8 | 19.5 | 41 | | 8 | 19.5 | NS | |
| Solomon Islands[44] | 1997 | 226 | | 91 | 40.3 | 226 | | 91 | 40.3 | 25% | |
| Taiwan[45] | 1998 | 3,029 | | 72 | 2.4 | 3,029 | | 72 | 2.4 | 1.40% | |
| Malaysia[46] | 1998^ | 356 | | 24 | 6.7 | NS | | NS | NS | 11.90% | |
| Samoa[47] | 1998^ | 8,767 | | 3,822 | 43.6 | 8,767 | | 3822 | 43.6 | 4.90% | |
| Kenya[48] | 1999 | 4,961 | | 563 | 11.3 | 4,961 | | 563 | 11.3 | 8.00% | |
| Australia*[49] | 2000 | 217 | | 49 | 22.6 | 217 | | 49 | 22.6 | 35% | |
| Australia*[50] | 2000 | 121 | | 80 | 66.1 | 121 | | 80 | 66.1 | 5.0% | |
| India[51] | 2000 | 4,249 | | 28 | 0.7 | 4,249 | | 28 | 0.7 | NS | |
| Egypt[52] | 2001 | 636 | | 99 | 15.6 | 636 | | 99 | 15.6 | 0.50% | |
| Egypt[52] | 2001 | 720 | | 6 | 0.8 | 720 | | 6 | 0.8 | 0.10% | |
| India[53] | 2001 | 12,586 | | 925 | 7.3 | 12,586 | | 925 | 7.3 | 5% | |
| Mali[54] | 2001 | 1,729 | | 310 | 17.9 | 1,729 | | 310 | 17.9 | 1.30% | |
| Mali[54] | 2002 | 1,632 | | 304 | 18.6 | 1,632 | | 304 | 18.6 | 0.70% | |
| Turkey[55] | 2002^ | 785 | | 16 | 2.0 | 785 | | 16 | 2.0 | 2.16% | |
| Tanzania[56] | 2003 | 820 | | 69 | 8.4 | 820 | | 69 | 8.4 | 1.50% | |
| Fiji[57] | 2004 | 258 | | 6 | 2.3 | 258 | | 6 | 2.3 | 32.6% | |
| Australia*[58] | 2004 | 582 | | 266 | 45.7 | 582 | | 266 | 45.7 | 16.10% | |
| Ghana[59] | 2004 | 463 | | 20 | 4.3 | 463 | | 20 | 4.3 | 0.00% | |
| Gabon[59] | 2005 | 454 | | 7 | 1.5 | 454 | | 7 | 1.5 | 0.70% | |
| Fiji[60] | 2006 | 3,462 | | 1259 | 36.4 | 3,462 | | 1259 | 36.4 | 18.50% | |
| Fiji[60] | 2006 | 451 | | 60 | 13.3 | 451 | | 60 | 13.3 | 14% | |
| Timor Leste[61] | 2007 | 1,535 | | 112 | 7.3 | 728 | | 82 | 11.3 | 17% | |
| Ghana[59] | 2007 | 1,394 | | 81 | 5.8 | 1,394 | | 81 | 5.8 | 0.10% | |
| Rwanda[59] | 2007 | 2,528 | | 32 | 1.3 | 2,528 | | 32 | 1.3 | 0.04% | |
| Nepal[62] | 2008^ | 878 | | 54 | 6.2 | NS | | NS | NS | 3.40% | |
| Nigeria[63] | 2009^ | 1,415 | | 14 | 1.0 | 1,415 | | 14 | 1.0 | 0.60% | |
| Australia*[58] | 2009^ | 2,001 | | 804 | 40.2 | 2,001 | | 804 | 40.2 | NS | |
| Cameroon[64] | 2010 | 400 | | 43 | 10.8 | 48 | | 2 | 4.2 | 1.80% | |
| Ethiopia[65] | 2010^ | 1,104 | | 29 | 2.6 | 1,104 | | 29 | 2.6 | 0.30% | |
| Tanzania[66] | 2010^ | 420 | | 17 | 4.0 | 393 | | 16 | 4.1 | 1.40% | |

*Resource-poor populations within high-income OECD countries, ^reflects year of publication when year study was commenced is unknown.

References

1. Chuttani CS, Pal SC (1971) An epidemiological study of streptococcal skin infection. Indian J Med Res 59: 1822-1826.

2. Dajani AS, Ferrieri P, Wannamaker L (1973) Endemic superficial pyoderma in children. Arch Dermatol 108: 517-522.

3. Nelson KE, Bisno AL, Waytz P, Brunt J, Moses VK, et al. (1976) The epidemiology and natural history of streptococcal pyoderma: an endemic disease of the rural southern United States. Am J Epidemiol 103: 270-283.

4. Taplin D, Lansdell L, Allen AM, Rodriguez R, Cortes A (1973) Prevalence of streptococcal pyoderma in relation to climate and hygiene. Lancet 1: 501-503.

5. Allen AM, Taplin D (1974) Skin infections in eastern Panama. Survey of two representative communities. Am J Trop Med Hyg 23: 950-956.

6. Koshi G, Benjamin V (1977) Surveillance of streptococcal infections in children in a south Indian community--a pilot survey. Indian J Med Res 66: 379-388.

7. Masawe AE, Nsanzumuhire H (1975) Scabies and other skin diseases in pre-school children in Ujamaa villages in Tanzania. Trop Geogr Med 27: 288-294.

8. Lines DR (1977) An Auckland high school health survey. Aust N Z J Med 7: 143-147.

9. Bechelli LM, Haddad N, Pimenta WP, Pagnano PM, Melchior E, Jr., et al. (1981) Epidemiological survey of skin diseases in schoolchildren living in the Purus Valley (Acre State, Amazonia, Brazil). Dermatologica 163: 78-93.

10. Belcher DW, Afoakwa SN, Osei-Tutu E, Wurapa FK, Osei L (1977) Endemic pyoderma in Ghana: a survey in rural villages. Trans R Soc Trop Med Hyg 71: 204-209.

11. Masawe AE, Nsanzumuhire H, Mhalu F (1975) Bacterial skin infections in preschool and school children in coastal Tanzania. Arch Dermatol 111: 1312-1316.

12. Lawrence DN, Facklam RR, Sottnek FO, Hancock GA, Neel JV, et al. (1979) Epidemiologic studies among Amerindian populations of Amazonia. I. Pyoderma: prevalence and associated pathogens. Am J Trop Med Hyg 28: 548-558.

13. Brahmadathan KN, Koshi G (1988) Epidemiology of streptococcal pyoderma in an orphanage community of a tropical country. J Trop Med Hyg 91: 306-314.

14. Bhavsar BS, Mehta RN (1985) Pyoderma--an epidemiological study through school survey in Surat District (South Gujarat). Indian J Public Health 29: 233-237.

15. Porter MJ (1980) Seasonal change and its effect on the prevalence of infectious skin disease in a Gambian village. Trans R Soc Trop Med Hyg 74: 162-168.

16. Margolis HS, Lum MK, Bender TR, Elliott SL, Fitzgerald MA, et al. (1980) Acute glomerulonephritis and streptococcal skin lesions in Eskimo children. Am J Dis Child 134: 681-685.

17. Patel RB, Udani RH, Khanna SA (1982) Pediatric dermatoses and eradication in slums. Indian J Pediatr 49: 135-139.

18. Sharma RS, Mishra RS, Pal D, Gupta JP, Dutta M, et al. (1984) An epidemiological study of scabies in a rural community in India. Ann Trop Med Parasitol 78: 157-164.

19. Porter MJ, Mack RW, Chaudhary MA (1984) Pediatric skin disease in Pakistan. A study of three Punjab villages. Int J Dermatol 23: 613-616.

20. Bhalla KK (1984) Pattern of skin diseases in a semi-urban community in Delhi area. Indian Journal of Dermatology, Venereology and Leprology 50: 213 - 214.

21. Jinadu MK (1985) Pediculosis humanus capitis among primary school children in Ile-Ife, Nigeria. J R Soc Health 105: 25-27.

22. Stenderup A SE, Mataika J (1983) Dermatomycosis and other skin diseases in a Fijian village. Fiji Medical Journal: 18 - 19.

23. Nicolle LE, Postl B, Urias B, Law B, Ling N (1990) Group A streptococcal pharyngeal carriage, pharyngitis, and impetigo in two northern Canadian native communities. Clin Invest Med 13: 99-106.

24. Eason RJ, Tasman-Jones T (1985) Resurgent yaws and other skin diseases in the Western Province of the Solomon Islands. P N G Med J 28: 247-250.

25. Sharma NK, Garg BK, Goel M (1986) Pattern of Skin Diseases in Urban School Children. Indian Journal of Dermatology, Venereology and Leprology 52: 330 - 331.

26. Bhatia V (1997) Extent and pattern of paediatric dermatoses in rural areas of central India. Indian J Dermatol Venereol Leprol 63: 22-25.

27. Harris M, Nako D, Hopkins T, Powell DM, Kenny C, et al. (1992) Skin infections in Tanna, Vanuatu in 1989. P N G Med J 35: 137-143.

28. Dagnew MB, Erwin G (1991) Epidemiology of common transmissible skin diseases among primary school children in north-west Ethiopia. Trop Geogr Med 43: 152-155.

29. Nimmo GR, Tinniswood RD, Nuttall N, Baker GM, McDonald B (1992) Group A streptococcal infection in an aboriginal community. Med J Aust 157: 521-522.

30. Henderson CA (1996) Skin disease in rural Tanzania. Int J Dermatol 35: 640-642.

31. Kottenhahn RK, Heck JE (1994) Prevalence of paediatric skin diseases in rural Honduras. Trop Doct 24: 87-88.

32. Figueroa JI, Fuller LC, Abraha A, Hay RJ (1996) The prevalence of skin disease among school children in rural Ethiopia--a preliminary assessment of dermatologic needs. Pediatr Dermatol 13: 378-381.

33. Van Buynder PG, Gaggin JA, Martin D, Pugsley D, Mathews JD (1992) Streptococcal infection and renal disease markers in Australian aboriginal children. Med J Aust 156: 537-540.

34. Streeton CL, Hanna JN, Messer RD, Merianos A (1995) An epidemic of acute post-streptococcal glomerulonephritis among aboriginal children. J Paediatr Child Health 31: 245-248.

35. Mahe A, Prual A, Konate M, Bobin P (1995) Skin diseases of children in Mali: a public health problem. Trans R Soc Trop Med Hyg 89: 467-470.

36. Schmeller W (1998) Community health workers reduce skin diseases in East African children. Int J Dermatol 37: 370-377.

37. San Sebastian M, Santi S (1999) The health status of rural school children in the Amazon basin of Ecuador. J Trop Pediatr 45: 379-382.

38. Gibbs S (1996) Skin disease and socioeconomic conditions in rural Africa: Tanzania. Int J Dermatol 35: 633-639.

39. Carapetis JR, Johnston F, Nadjamerrek J, Kairupan J (1995) Skin sores in Aboriginal children. J Paediatr Child Health 31: 563.

40. Carapetis JR, Connors C, Yarmirr D, Krause V, Currie BJ (1997) Success of a scabies control program in an Australian aboriginal community. Pediatr Infect Dis J 16: 494-499.

41. Fry AM, Jha HC, Lietman TM, Chaudhary JS, Bhatta RC, et al. (2002) Adverse and beneficial secondary effects of mass treatment with azithromycin to eliminate blindness due to trachoma in Nepal. Clin Infect Dis 35: 395-402.

42. Shelby-James TM, Leach AJ, Carapetis JR, Currie BJ, Mathews JD (2002) Impact of single dose azithromycin on group A streptococci in the upper respiratory tract and skin of Aboriginal children. Pediatr Infect Dis J 21: 375-380.

43. Norazah A, Normaznah Y, Kamel AG, Rohani MY (1995) Streptococcal impetigo among aboriginal children in Malaysia. Southeast Asian J Trop Med Public Health 26: 803-804.

44. Lawrence G, Leafasia J, Sheridan J, Hills S, Wate J, et al. (2005) Control of scabies, skin sores and haematuria in children in the Solomon Islands: another role for ivermectin. Bull World Health Organ 83: 34-42.

45. Wu YH, Su HY, Hsieh YJ (2000) Survey of infectious skin diseases and skin infestations among primary school students of Taitung County, eastern Taiwan. J Formos Med Assoc 99: 128-134.

46. Norhayati binti Moktar M, Noor Hayati MI, Nor Fariza N, Rohani AK, Halimah AS, et al. (1998) Health status of Orang Asli (aborigine) community in Pos Piah, Sungai Siput, Perak, Malaysia. Southeast Asian J Trop Med Public Health 29: 58-61.

47. Steer AC, Adams J, Carlin J, Nolan T, Shann F (1999) Rheumatic heart disease in school children in Samoa. Arch Dis Child 81: 372.

48. Schmeller W, Dzikus A (2001) Skin diseases in children in rural Kenya: long-term results of a dermatology project within the primary health care system. Br J Dermatol 144: 118-124.

49. Wong LC, Amega B, Connors C, Barker R, Dulla ME, et al. (2001) Outcome of an interventional program for scabies in an Indigenous community. Med J Aust 175: 367-370.

50. Lehmann D, Tennant MT, Silva DT, McAullay D, Lannigan F, et al. (2003) Benefits of swimming pools in two remote Aboriginal communities in Western Australia: intervention study. BMJ 327: 415-419.

51. Kumar R, Vohra H, Chakraborty A, Sharma YP, Bandhopadhya S, et al. (2009) Epidemiology of group A streptococcal pharyngitis & impetigo: a cross-sectional & follow up study in a rural community of northern India. Indian J Med Res 130: 765-771.

52. Fathy H, El-Mongy S, Baker NI, Abdel-Azim Z, El-Gilany A (2004) Prevalence of skin diseases among students with disabilities in Mansoura, Egypt. East Mediterr Health J 10: 416-424.

53. Dogra S, Kumar B (2003) Epidemiology of skin diseases in school children: a study from northern India. Pediatr Dermatol 20: 470-473.

54. Faye O, N'Diaye HT, Keita S, Traore AK, Hay RJ, et al. (2005) High prevalence of non-leprotic hypochromic patches among children in a rural area of Mali, West Africa. Lepr Rev 76: 144-146.

55. Inanir I, Sahin MT, Gunduz K, Dinc G, Turel A, et al. (2002) Prevalence of skin conditions in primary school children in Turkey: differences based on socioeconomic factors. Pediatr Dermatol 19: 307-311.

56. Ferie J, Dinkela A, Mbata M, Idindili B, Schmid-Grendelmeier P, et al. (2006) Skin disorders among school children in rural Tanzania and an assessment of therapeutic needs. Trop Doct 36: 219-221.

57. Thomas M, Woodfield G, Moses C, Amos G (2005) Soil-transmitted helminth infection, skin infection, anaemia, and growth retardation in schoolchildren of Taveuni Island, Fiji. N Z Med J 118: U1492.

58. Andrews RM, Kearns T, Connors C, Parker C, Carville K, et al. (2009) A regional initiative to reduce skin infections amongst aboriginal children living in remote communities of the Northern Territory, Australia. PLoS Negl Trop Dis 3: e554.

59. Hogewoning A, Amoah A, Bavinck JN, Boakye D, Yazdanbakhsh M, et al. (2013) Skin diseases among schoolchildren in Ghana, Gabon, and Rwanda. Int J Dermatol 52: 589-600.

60. Steer AC, Jenney AW, Kado J, Batzloff MR, La Vincente S, et al. (2009) High burden of impetigo and scabies in a tropical country. PLoS Negl Trop Dis 3: e467.

61. dos Santos MM, Amaral S, Harmen SP, Joseph HM, Fernandes JL, et al. (2010) The prevalence of common skin infections in four districts in Timor-Leste: a cross sectional survey. BMC Infect Dis 10: 61.

62. Walker SL, Shah M, Hubbard VG, Pradhan HM, Ghimire M (2008) Skin disease is common in rural Nepal: results of a point prevalence study. Br J Dermatol 158: 334-338.

63. Ogunbiyi AO, Omigbodun Y, Owoaje E (2009) Prevalence of skin disorders in school children in southwest Nigeria. Int J Adolesc Med Health 21: 235-241.

64. Bissek AC, Tabah EN, Kouotou E, Sini V, Yepnjio FN, et al. (2012) The spectrum of skin diseases in a rural setting in Cameroon (sub-Saharan Africa). BMC Dermatol 12: 7.

65. Murgia V, Bilcha KD, Shibeshi D (2010) Community dermatology in Debre Markos: an attempt to define children's dermatological needs in a rural area of Ethiopia. Int J Dermatol 49: 666-671.

66. Komba EV, Mgonda YM (2010) The spectrum of dermatological disorders among primary school children in Dar es Salaam. BMC Public Health 10: 765.
